# The impact of semi-upright position on severity of sleep disordered breathing in patients with obstructive sleep apnea: a two-arm, prospective, randomized controlled trial

**DOI:** 10.1186/s12871-023-02193-y

**Published:** 2023-07-13

**Authors:** Gincy A. Lukachan, Azadeh Yadollahi, Dennis Auckley, Bojan Gavrilovic, John Matelski, Frances Chung, Mandeep Singh

**Affiliations:** 1 Department of Anesthesia, Believers Church Medical College Hospital, Thiruvalla, Kerala India; 2 KITE - Toronto Rehabilitation Institute, University Health Network, University of Toronto, Toronto, ON Canada; 3 Division of Pulmonary, Critical Care and Sleep Medicine, MetroHealth Medical Center, Case Western Reserve University, Cleveland, OH USA; 4 Biostatistics Research Unit, University Health Network, Toronto, ON Canada; 5 Department of Anesthesia, Toronto Western Hospital, University Health Network, University of Toronto, 399 Bathurst Street, McL 2-405, Toronto, ON M5T 2S8 Canada

**Keywords:** Obstructive sleep apnea, Supine-related OSA, Surgery, Elevated position, Positional therapy

## Abstract

**Background:**

The severity of sleep-disordered breathing is known to worsen postoperatively and is associated with increased cardio-pulmonary complications and increased resource implications. In the general population, the semi-upright position has been used in the management of OSA. We hypothesized that the use of a semi-upright position versus a non-elevated position will reduce postoperative worsening of OSA in patients undergoing non-cardiac surgeries.

**Methods:**

This study was conducted as a prospective randomized controlled trial of perioperative patients, undergoing elective non-cardiac inpatient surgeries. Patients underwent a preoperative sleep study using a portable polysomnography device. Patients with OSA (apnea hypopnea index (AHI) > 5 events/hr), underwent a sleep study on postoperative night 2 (N2) after being randomized into an intervention group (Group I): semi-upright position (30 to 45 degrees incline), or a control group (Group C) (zero degrees from horizontal). The primary outcome was postoperative AHI on N2. The secondary outcomes were obstructive apnea index (OAI), central apnea index (CAI), hypopnea index (HI), obstructive apnea hypopnea index (OAHI) and oxygenation parameters.

**Results:**

Thirty-five patients were included. Twenty**-**one patients were assigned to the Group 1 (females-14 (67%); mean age 65 ± 12) while there were fourteen patients in the Group C (females-5 (36%); mean age 63 ± 10). The semi-upright position resulted in a significant reduction in OAI in the intervention arm (Group C vs Group I postop AHI: 16.6 ± 19.0 vs 8.6 ± 11.2 events/hr; overall *p* = 0.01), but there were no significant differences in the overall AHI or other parameters between the two groups. Subgroup analysis of patients with “supine related OSA” revealed a decreasing trend in postoperative AHI with semi-upright position, but the sample size was too small to evaluate statistical significance.

**Conclusion:**

In patients with newly diagnosed OSA, the semi-upright position resulted in improvement in obstructive apneas, but not the overall AHI.

**Trial registration:**

This trial was retrospectively registered in clinicaltrials.gov NCT02152202 on 02/06/2014.

**Supplementary Information:**

The online version contains supplementary material available at 10.1186/s12871-023-02193-y.

## Introduction

Obstructive sleep apnea (OSA) is a common sleep-related breathing disorder, associated with increased morbidity and mortality in the general and surgical population [1, 2] in the perioperative period [3]. It is an independent risk-factor for post-operative cardiac and respiratory complications [4–7] resulting in increased utilization of health care resources [8].

The screening and treatment of OSA is found to be cost effective on the lifetime horizon [9]. According to the current guidelines adult patients at risk of OSA should be screened preoperatively using validated tools such as STOP-Bang, P-SAP, Berlin, and ASA Check List [10]. The American Society of Anesthesiology (ASA) practice guidelines on the perioperative management of OSA advice to consider the initiation of continuous positive airway pressure (CPAP) therapy preoperatively in patients with newly detected severe OSA. [11], Despite improvement in OSA severity and oxygenation with CPAP, poor patient compliance has been a hurdle to their use in the perioperative period [12, 13]. Other alternatives to OSA treatment, such as weight reduction, [14] custom-made orthodontic appliances [15], and surgery (orthodontic surgery, [16] uvulopalatopharyngeoplasty, [17] tonsillectomy, [18] or bariatric surgery [19]) are not feasible in the preoperative period. There is a need for alternative approaches for the management of OSA in surgical patients.

Positional therapy could be a useful intervention in surgical patients with OSA in the perioperative period [20]. This option may be more feasible in the postoperative setting as it is cost-effective, easy to administer, and can be adjusted to allow patient comfort. In the general population, sleeping in the non-supine and elevated posture was found to be effective in reducing OSA severity [21–23]. The utility of positional therapy may be greater in patients with supine-related OSA. Supine-related OSA is defined as Apnea–hypopnea index (AHI) > 5 events/hr, and where the supine AHI was more than twice the AHI of the non-supine AHI, and the non-supine AHI was less than 5 events/hr [24, 25].

The American Society of Anesthesiology practice guidelines on the perioperative management of OSA recognized that positional therapy may improve the AHI in patients with OSA, but acknowledged that the literature was “insufficient to evaluate the effects of positioning adult OSA patients in the postoperative setting” [11]. We hypothesized that the use of a semi-upright position versus a supine position will prevent postoperative worsening of OSA in patients undergoing non-cardiac surgeries. The objective of the study was to determine whether a semi-upright versus supine position while asleep helps decrease the postoperative worsening of AHI in surgical patients with newly diagnosed OSA. The secondary objective was to study the impact of the semi-upright position in a subgroup of patients with supine-related OSA.

## Methods

### Study design

This was a two-arm, prospective, randomized controlled, proof of concept trial. The intervention was patient positioning in a semi-upright position (Group I: intervention, head-end elevation 30 to 45 degrees from horizontal), compared to supine position (Group C: control).

### Study setting

This study was conducted at Toronto Western Hospital and Mount Sinai Hospital in Toronto, over a period of seven months. Institutional Review Board approval was obtained from both hospitals prior to start of this study (University Health Network 11-0056AE and Mount Sinai Hospital 11–0021-E). This trial was registered at www.clincialtrials.gov (NCT02152202).

The inclusion criteria of patients were adult patients, American Society of Anesthesiologists (ASA) physical status I to IV, undergoing elective inpatient non-cardiac surgery with newly diagnosed OSA. The exclusion criteria were: patients with OSA on treatment (continuous positive airway pressure (CPAP), oral appliance, or previous OSA surgery); known cervical, shoulder, spine abnormalities, and/or chronic pain predisposing to difficulty in maintaining a sitting position and specific types of surgery, such as hip or spine, where a sitting position would be contraindicated postoperatively. Patients were screened by using the STOP-Bang questionnaire [26]. Patients identified as high risk of OSA (STOP-Bang score of three or greater) were consented to undergo a home portable polysomnography (PSG) and OSA status was confirmed by an AHI over 5 events per hour.

### Patient recruitment, intervention and follow-up

Portable PSG was performed using a 10-channel portable PSG device (Embletta X100; Embla, Broomfield, CO). The PSG was obtained preoperatively (preop) at home and on postoperatively (postop) on N 2 [27]. The Embletta X100 is a level 2 diagnostic tool for OSA and has been validated against laboratory PSG [27]. The PSG recording montage comprised two electroencephalographic channels (C3 and C4), left or right electro-oculogram, chin muscle electromyogram, nasal cannula (pressure), thoracic and abdominal respiratory effort bands, body-position sensor, and pulse oximetry. At bedtime, the portable PSG device was connected to the patient by a PSG technician at their home. Patients were taught how to disconnect the device, which was picked up by the same sleep technician the following morning. The portable PSG recording was scored by a certified PSG technologist who was supervised by a sleep physician.

Apneas were defined as a reduction in airflow from intranasal pressure of at least 90% for 10 s or longer, and hypopneas as reduction in flow of at least 30% for 10 s or longer, associated with ≥ 4% oxygen desaturation [28]. Apneas and hypopneas were classified as either obstructive (presence of breathing effort) or central (absence of breathing effort) events. Mixed apneas were classified for events that began as central for at least 10 s and ended as obstructive, with a minimum of three obstructive efforts. AHI was the average number of apnea and hypopnea episodes per hour of recording. Apnea index was calculated as the average number of apnea episodes per hour. Hypopnea index was the average number of hypopnea episodes per hour. The secondary outcomes were obstructive apnea index (OAI), calculated as the total number of obstructive apneas divided total sleep time (TST); central apnea index (CAI) calculated as the total number of central apneas per hour; obstructive apnea hypopnea index (OAHI), calculated as the total number of obstructive apneas and hypopneas per hour; oxygen desaturation index (ODI), number of events with oxygen desaturation below 4% threshold per hour, and CT90, cumulative percentage of sleep duration with oxygen desaturation less than 90%.

### Randomization and allocation concealment

Patients with OSA (defined as AHI > 5 events/hr), were randomized into two groups: Control or Intervention groups (computer generated blocks of 8) by the research analyst, who was not involved in group allocation or data collection during the study. Group allocation was concealed using sealed, opaque envelops, and patients were assigned to their group following surgery. In the Control group (Group C), there was no bed elevation, and patients were positioned at bedtime with no head elevation or bed angle to zero degrees from the horizontal. In the Intervention group (Group I), patients were positioned at bedtime in a semi-upright position with bed elevated to 30 to 45 degrees from horizontal. The bed angle was measured by a research assistant using an in-built bed angle monitor, or a goniometer, wherever applicable. The bed angle measurements were performed at night and in the morning to monitor compliance with the allocated bed position. Patients had the option to request changing the bed angle to facilitate recovery from surgery in view of pain and discomfort.

### Perioperative anesthetic care and postoperative pain management

A standardized balanced anesthetic technique was used in all patients per routine care. In general anesthesia (GA), patients received an induction dose of propofol, opioid (fentanyl and/or hydromorphone), an inhalational agent (sevoflurane or desflurane), and a muscle relaxant (rocuronium). The muscle relaxant was reversed with neostigmine and atropine. In regional anesthesia (RA), patients received spinal anesthetic and sedation using midazolam, fentanyl and a propofol infusion (20–150 mcg/kg/min). Use of intrathecal opioid (100 mcg preservative free morphine) was at the discretion of the anesthesiologist. Both groups received intravenous or oral narcotics in the postoperative period guided by the Acute Pain Service team, as per our institutional standard of care. Pain was evaluated on a score of 0–10, with 0 as no pain and 10 as the most excruciating pain. Intravenous morphine by patient-controlled analgesia was initiated when the verbal pain score was 4 or higher. The research assistant visited patients daily to assist them with application and removal of the portable PSG, collect data, and document adverse events during the hospital stay.

### Target sample size

The primary outcome of our study was AHI on postop N2. There was no previous research from the perioperative setting evaluating the impact of body positioning. Previous studies in the general population found that the mean change in AHI between the upright position (6 ± 12 events/hr), and no head elevation (29 ± 6 events/hr), respectively [23, 29]. The original sample size calculated in the protocol was 28 in each arm calculated after taking a minimal clinically significant difference (MCSD) of an effect size (change in AHI) of at least 10 events per hour from baseline, a power of 0.9 and a standard deviation of 10, after adjusting for an estimated drop-out, and loss of follow up to a total of 20%. However, the study was terminated early due to concerns with funding, and a final sample size of 32 patients was obtained, which had sufficient power of 0.8, with type 1 error of 0.05. It was decided to proceed with analysis of the data by the senior authors.

### Statistical analysis

Analyses were performed using the SAS 9.2 statistical software for Windows (SAS Institute, Cary, NC) or R (version 3.1.1) [30]. The analysis was blinded to allocation until the completion of data accrual period. Because of patient preference and deviation from assignment of intervention, a per-protocol analysis was performed for this study, where patients were analyzed based on the bed angle monitor reading noted in the morning following their PSG to show no deviation from protocol. An intention-to-treat analysis was performed as sensitivity analysis, meaning that all participants were analyzed in the group to which they were randomized.

Baseline demographic variables are summarized for the entire study population and by treatment group using standard bivariate methods, as implemented in R package tableone [31]. For each variable we include a standardized mean difference, along with a p-value against the null hypothesis of equality between groups.

Continuous variables were compared using two-tailed, paired t-tests for variables with normally distributed data and Wilcoxon signed rank test for variables with non-normally distributed data.

Pre-defined linear regression was performed for the primary and secondary outcomes, with preop AHI and supine-related OSA as covariates. Supine-related OSA was defined as AHI > 5 events/hr, and where the supine AHI was more than twice the AHI of the non-supine AHI, and the non-supine AHI was less than 5 events/hr [24]. A two-sided *p* value < 0.05 was considered significant and controlled for repeated observations, wherever applicable.

## Results

### Study population

**Fig. 1:**
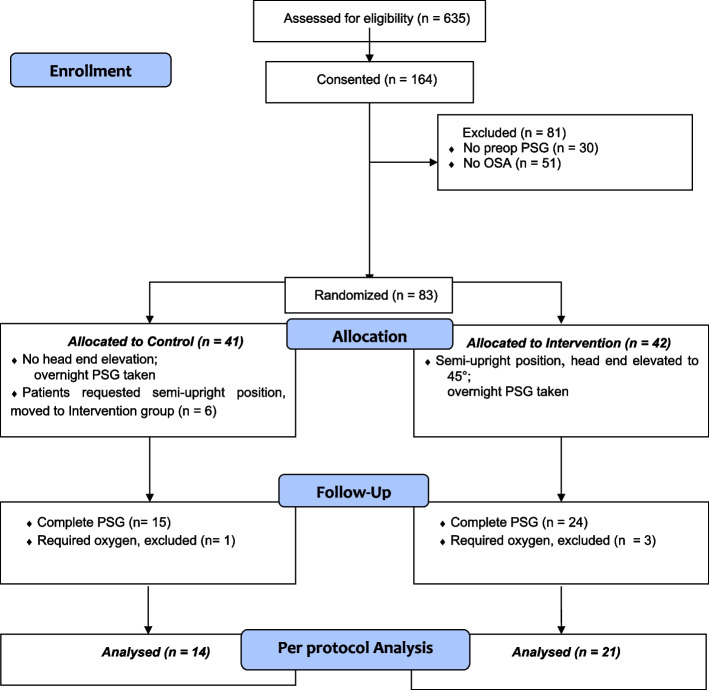
Participant flow in the study

Patient recruitment and flow is summarized in Fig. 1, based on the CONSORT recommendations. A total of 635 patients were screened preoperatively, with 164 patients giving consent, of which 135 patients completed home PSG study. Eighty-three patients with OSA (AHI > 5 events/hr) were randomized, Group C: 41 and Group I: 42. During the study, six patients in Group C requested to change position to semi-upright position and were allocated to Group I. Complete postoperative N2 PSG data were obtained from 15 and 24 patients for Group C and I, respectively. This was partly because of patient refusal to undergo the PSG postoperatively while recovering from surgery, primarily due to postoperative pain and discomfort. Four patients were excluded as they required oxygen supplementation. Per protocol analysis was done for 14 patients in Group C and 21 patients in Group I. The baseline characteristics for PP and ITT analysis are presented in Table 1, and Supplementary table 1, respectively. While randomization led to a more balanced distribution of baseline demographic variables, deviation of protocol resulted in disturbances for the PP analysis where control group had higher neck circumference and lower OAI and CT 90 values (SMD > 0.8). The preop PSG (Table 1) data showed no difference in AHI, OAHI, HI between Group C and Group I. The baseline OAI was significantly higher (11.7 ± 9.2 vs. 6.0 ± 3.6 events per hour; *p* = 0.01) while CAI was significantly lower (0.7 ± 1.4 vs 3.7 ± 9.5; *p* = 0.04) in Group I than Group C. The CT90 was significantly higher (3.8 (0.4—6.1) % vs. 0.7 (0.1—1.2) %; *p* = 0.04) and the lowest SaO_2_ was significantly lower (79.1 ± 6.1% vs 83.2 ± 5.0%; *p* = 0.04) in Group I vs Group C.

**Table 1:** Patient demographics across the two groups (Per-protocol)

	Control (*n* = 14)	Intervention (*n* = 21)	*P* value	Standardized Mean Difference
**Age** (years)	63 ± 10	65 ± 12	0.60	0.188
**BMI** (kg/m^2^)	32.8 ± 4	35.2 ± 7	0.26	0.420
**Neck Circumference** (cm)	43.3 ± 3	41.0 ± 3	0.04	0.752
**Gender F/M**	5/9	14/7	0.09	0.651
**STOP-Bang score**	4.5 ± 1	4.5 ± 1	0.95	0.020
**Co-morbidity**				
Hypertension	7	12	0.74	0.144
Gastroesophageal reflux	2	7	0.26	0.459
Diabetes Mellitus	1	6	0.20	0.583
Smoker	2	2	1.00	0.147
Asthma	1	3	0.64	0.232
COPD	1	1	1.00	0.101
CAD	3	0	0.06	0.739
**Type of surgery**			0.65	0.707
Orthopedic	8	12		
General	5	6		
Gynecology	0	2		
Urology	1	0		
**Type of Anesthesia**			1.00	0.048
General	7	10		
Spinal	7	11		
**ASA Status**			0.16	0.589
II	8	7		
III	5	14		
**Total amount of opioids (in mg IV morphine equivalents)**				
1^st^ 24 h	10.3 [5.3—19.8]	11.0 [5.—20]	0.84	0.080
1^st^ 48 h	21.4 [13.3 – 44.4]	33.4 [15.0—55.0]	0.45	0.186
1^st^ 72 h	25.8 [14.2—60.50]	41.4 [25.0—81.5]	0.29	0.259
**Preoperative sleep study data between the two groups**				
AHI	18.1 ± 13.3	21.4 ± 13.1	0.48	0.246
OAI	6.1 ± 3.7	11.7 ± 9.2	0.04	0.807
OAHI	14.6 ± 7.2	20.7 ± 12.6	0.11	0.593
CAI	3.7 ± 9.5	0.7 ± 1.4	0.16	0.444
HI	8.6 ± 5.2	9.0 ± 7.3	0.84	0.071
ODI	15.5 [13.5—23.7]	17.9 [12.1—39.1]	0.57	0.281
CT90	0.7 [0.1—1.2]	3.8 [0.4 – 6.1]	0.04	0.804
Average SaO2	93.4 ± 1.27	93.1 ± 2.4	0.71	0.137
Lowest SaO2	83.2 ± 5.0	79.1 ± 6.1	0.04	0.748

Data are expressed as mean (SD) or median (interquartile range IQR) where appropriate. Standardized Mean Difference was calculated using R package tableone

*BMI* body mass index, *COPD* chronic obstructive pulmonary disease, *CAD* coronary artery disease, *OR* operating room, *h* hours, *AHI* Apnea–Hypopnea Index, Apnea index average number of apnea episodes per hour, *Arousal \index* number of arousals × 60 / Total sleep time, *CAI* Central Apnea Index: total number of central apneas per hour, *CT90* cumulative percentage of Total sleep time with oxygen desaturation below 90%, *HI* Hypopnea index, average number of hypopnea episodes per hour, *OAHI* Obstructive Apnea Hypopnea Index, total number of obstructive apneas and hypopneas per hour, *OAI* Obstructive Apnea Index: total number of obstructive apneas divided by Total sleep time, *ODI* Oxygen Desaturation Index, number of events with oxygen desaturation below 4% threshold in one hour, *REM%* Time spent in rapid eye movement stage of sleep, *SaO*_*2*_ saturation of oxygen in hemoglobin

### Primary outcome

**Fig. 2:**
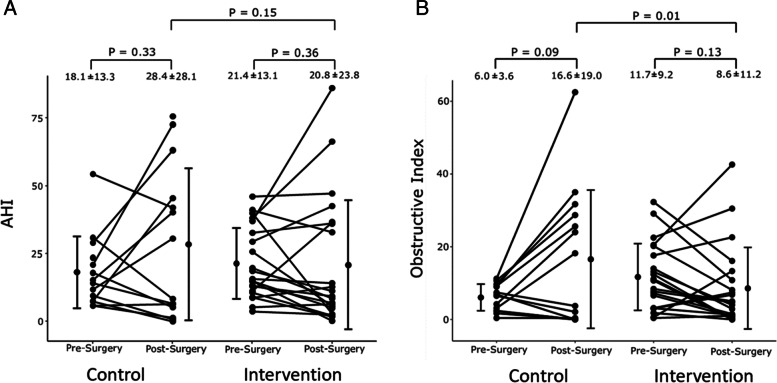
A. The effect of semi-upright position on apnea–hypopnea index in the two groups per-protocol analysis. B. The effect of semi-upright position on obstructive index in the two groups per-protocol analysis

Comparing postop N2 vs preop baseline, the AHI increased in Group C while it decreased in Group I (Table 2). The differences were not significant within the groups or between groups. (Group C: postop AHI vs preop AHI: 28.4 ± 28.1 vs 18.1 ± 13.3 events/hr, *p* = 0.33; Group I: postop AHI vs preop AHI: 20.8 ± 23.8 vs 21.4 ± 13.1; *p* = 0.36); overall *p* = 0.15. (Fig. 2A).

**Table 2:** Postoperative sleep-related outcomes in both groups

**Variable**	Control (*n* = 14)			Intervention (*n* = 21)			Between group comparison (ANCOVA)
	Pre-op	Post-op	*p*-value	Pre-op	Post-op	*p*-value	*p*-value
AHI	18.1 ± 13.3	28.4 ± 28.1	0.33	21.4 ± 13.1	20.8 ± 23.8	0.36	0.15
OAI	6.0 ± 3.6	16.6 ± 19.0	0.09	11.7 ± 9.2	8.6 ± 11.2	0.13	0.01^*^
CAI	3.7 ± 9.5	3.6 ± 4.9	0.35	0.7 ± 1.4	1.3 ± 3.5	0.79	0.11
HI	8.6 ± 5.2	8.6 ± 8.4	0.9	9.0 ± 7.3	11.3 ± 11.4	0.34	0.49
OAHI	14.6 ± 7.2	25.2 ± 24.2	0.14	20.7 ± 12.6	19.8 ± 21.5	0.54	0.09
ODI	19.3 ± 12.5	26.2 ± 24.7	0.39	23.1 ± 14.3	26.1 ± 27.1	0.66	0.55
CT90	1.3 ± 2.0	28.1 ± 35.4	0.003*	4.4 ± 4.9	15.7 ± 19.8	0.04^*^	0.11
Average SaO2	93.4 ± 1.3	90.0 ± 3.6	0.01*	93.1 ± 2.4	90.3 ± 3.8	0.002^*^	0.40
Lowest SaO2	83.2 ± 5.0	75.0 ± 21.2	0.26	79.0 ± 6.1	77.8 ± 9.9	0.79	0.91
Supine %	44.5 ± 30.3	86.8 ± 20.4	< 0.001	43.0 ± 35.6	66.2 ± 42.4	0.06	0.40

*AHI* Apnea–Hypopnea Index, *Apnea index* average number of apnea episodes per hour, *CAI* Central Apnea Index: total number of central apneas per hour, *CT90* cumulative percentage of total sleep time with oxygen desaturation below 90%, *HI* Hypopnea index, average number of hypopnea episodes per hour, *OAHI* Obstructive Apnea Hypopnea Index, total number of obstructive apneas and hypopneas per hour, *OAI* Obstructive Apnea Index: total number of obstructive apneas divided sleep time, *ODI* Oxygen Desaturation Index, number of events with oxygen desaturation below 4% threshold in one hour, *SaO*_*2*_ saturation of oxygen in hemoglobin. Statistically significant *P* values are indicated with an asterisk (*)

Comparing postop N2 vs preop parameters, the changes in OAI within the two groups were not significant but there was an overall significant change between the two groups (Group C vs Group I: postop AHI 16.6 ± 19.0 vs 8.6 ± 11.2 events/hr); (overall *p* = 0.01) (Fig. 2B). There were no significant differences in CAI, HI, OAHI within the two groups and between groups (Table 2).

Among the oxygenation parameters, CT90 (Supplementary Fig. 3) significantly increased postoperatively in both groups (Group C: postop CT90 vs preop: 4.4 ± 4.9% vs 1.3 ± 2.0%, *p* = 0.003; Group I: postop CT90 vs preop: 15.7 ± 19.8% vs 4.4 ± 4.9%; *p* = 0.04), and the average SaO_2_ were significantly decreased in the postoperative period for both groups (Group C: postop average SaO_2_ vs preop: 90.0 ± 3.6% vs 93.4 ± 1.3%, *p* = 0.01; Group I: postop average SaO_2_ vs preop: 90.3 ± 3.8% vs 93.1 ± 2.4%, *p* = 0.002). However, between group comparison did not show a significant difference (Table 2).

The other parameters were not significantly different from preop to postop and between the two groups (Table 2).

### Subgroup analysis

**Fig. 3:**
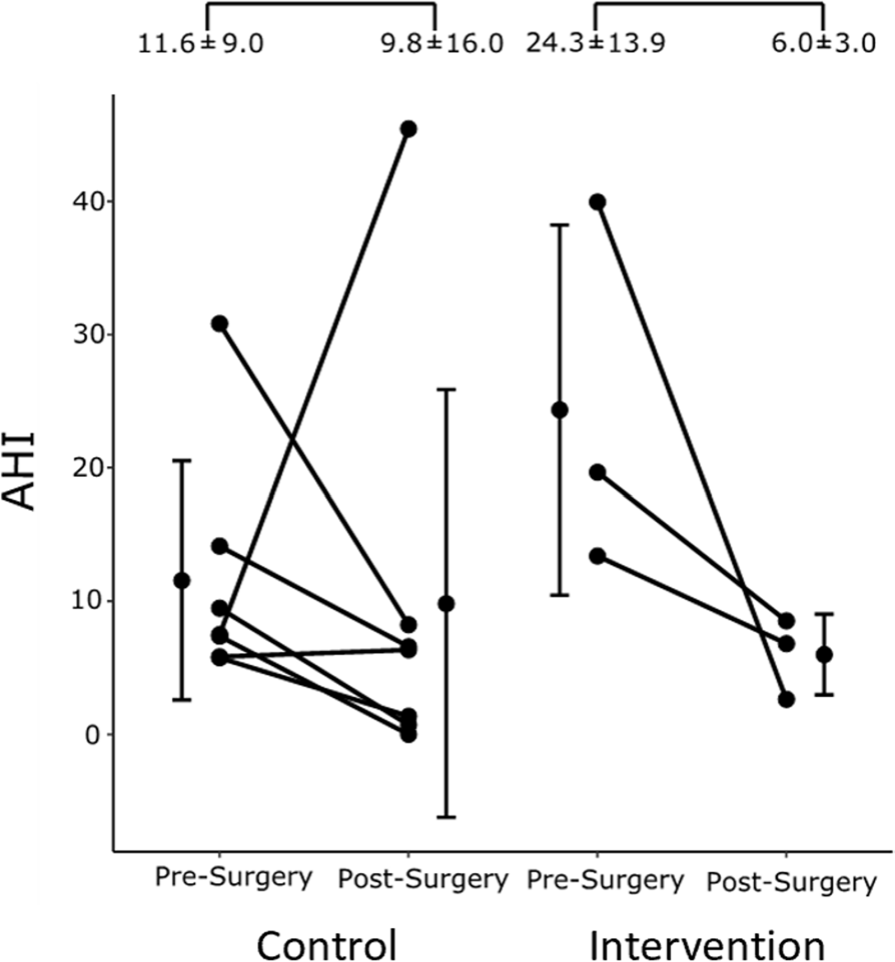
The effect of semi-upright body position on the apnea–hypopnea index (AHI) in patients with supine-related OSA (*n* = 10)

The impact of body position was examined in patients classified as “supine-related OSA” (*n* = 8; Group C: 5 patients, and Group I: 3 patients). There was greater reduction in mean AHI in Group I (postop AHI vs preop AHI: 6.0 ± 3.0 vs 24.3 ± 13.9 events/hr) than in Group C (Fig. 3), but the sample size was too small to evaluate statistical significance.

## Discussion

This is a novel study in the perioperative setting to evaluate the efficacy of semi-upright position postoperatively for management of newly diagnosed OSA. We found that it is feasible to institute positional therapy in the form of semi-upright position in the postoperative period for OSA patients. The elevated position resulted in a significant reduction in OAI by eight events per hour, but not the AHI, CAI, HI, and OAHI. The lack of positive results in AHI may be because the patients in the intervention group had significantly worse OAI, lower SaO_2,_ and higher CT 90 preoperatively. Nevertheless, in the surgical patients with supine-related OSA, we were able to demonstrate the effectiveness of semi-upright position as the mean AHI decreased by 18 events per hour.

Although positive airway pressure (PAP) is the mainstay of treatment for moderate to severe OSA, perioperative adherence has been poor as studies have demonstrated only 34% CPAP adherence [13] and 45% auto-titrated CPAP adherence [12] in patients with newly diagnosed OSA treated with PAP therapy before surgery. This suggests a need for alternative therapies for these patients. We found that positional therapy is a feasible alternative treatment option for OSA patients in the perioperative setting especially in those with supine related OSA. These findings could be explained by the close association of upper airway collapsibility with body, head, and neck positioning [24].

Previous work has suggested that the semi-upright position significantly enlarges the upper airway dimensions [32, 33]. The mean upper airway volume was greater with 44° head elevation compared to supine position [33]. Mild elevations of the head of the bed by 7.5° were associated with reductions in the AHI and improvements in oxygen parameters [34]. In a randomized crossover study of 30 postpartum women with OSA in the post-anesthesia care unit, 45° elevation of the upper body caused a significant reduction in AHI compared to non-elevated position [35]. A recently published randomized crossover trial among perioperative patients with moderate to severe OSA, compared high-flow nasal cannula (20 l/min with 40% oxygen concentration) with or without 30-degree head-of-bed elevation [36]. Patients were assessed with modified apnea hypopnea index, based exclusively on the airflow signal without arterial oxygen saturation criteria. High-flow nasal cannula caused significant improvement in OSA independently, with an additive effect when combined with 30-degree head-of-bed elevation (compared to Control flow-based AHI reduced by 10.9 (95% CI, 1 to 21) events · h–1, *P* = 0.028; and 23 (95% CI, 13 to 32) events · h–1, *P* < 0.001 respectively).

Body position may affect factors such as upper-airway passive collapsibility, airway dilator muscle activity, loop gain, and arousal threshold in patients with OSA [37]. These factors may play a role in how elevating the upper body can reduce OSA severity. In patients with OSA, pharyngeal critical closing pressure is higher in the supine position than lateral position [38] which may be related to reduction in functional residual capacity [39]. An increase in the diaphragmatic descent during the respiratory cycle leads to an increase in lung volume and thus an increase in longitudinal traction on the upper airway which increase upper airway caliber during sleep and anesthesia [40–42]. In addition, rostral fluid shift may worsen OSA severity and thus consolidate the effects of gravity on the propensity of OSA as demonstrated in healthy men [43, 44] and non-obese men [45].

A retrospective study on OSA patients with upper airway surgery found that the prevalence of positional OSA increased from 26 to 54% in those with persistent OSA at six months [46]. Among the non-responders to OSA surgery, almost 70% of patients were position dependent on preoperative PSG with no improvement at six months postoperatively [47]. This highlights the need to explore positional therapy, especially in those with positional OSA.

In a systematic review of positional therapy for OSA, CPAP was better than positional therapy to lower the AHI, while positional therapy was better than inactive controls to lower the AHI and improved daytime sleepiness [48]. Long term compliance and treatment benefit from positional therapy needs to be determined by longitudinal studies, and compared to other modalities such as CPAP.

In the general population, patients with supine-related OSA may be a suitable phenotype to benefit from positional therapy [24]. Good initial control of the OSA severity has been demonstrated but there is a lack of long-term compliance and outcome data [24]. The supine-related OSA phenotype can be easily identified on the preoperative sleep study. Though our data was limited, we found that patients with supine-related OSA benefit from postoperative semi-upright position with a reduction in AHI. Thus, we recommend the incorporation of semi-upright positioning as a practical adjunct to the perioperative management of OSA patients. It would be useful in those at high risk of OSA, newly diagnosed OSA or CPAP nonadherent patients. Further studies on custom-made pillows (to allow for elevated head position, or lateral position), tennis ball t-shirt, [21] or body position alarm devices [49] need to be done. Future studies aimed at localizing the site of obstruction by performing a drug induced sleep endoscopy or Point of care ultrasonography (POCUS) prior to randomization may also help to identify the subset of patients who will benefit with position therapy.

The limitations to our study were a small sample size with limited numbers in patients with supine-related OSA. Second, there can be variability in how the body position is reported and scored on the PSG. We used an accelerometer attached to the portable PSG which was placed on the patient′s chest. In-built automatic position sensors define body position as a categorical variable rather than a continuous variable, and may not reflect the physiological impact of various body positions on the collapsibility of the upper airway [24]. Third, head and neck position can independently influence the AHI [50]. Recording trunk position does not account for the effect of head and neck on upper-airway collapsibility and the impact on OSA severity [51, 52]. However, we were able to show that the elevated position resulted in a significant reduction in OAI by eight events per hour. In those with supine-related OSA, we found a non-significant decrease in the mean AHI by 18 events per hour.

## Conclusion

We found that the semi-upright position compared to supine position reduced postoperative OAI, indicating reduction in upper airway collapsibility and obstructive apneas. There was a decreasing trend in postoperative AHI in patients with supine-related OSA. Further studies on postoperative positional therapy are needed.

## Supplementary Information

**Additional file 1.**

## Data Availability

All data and materials in this manuscript are available from the corresponding author on reasonable request.
