# Supplementary material for: The impact of semi-upright position on severity of sleep disordered breathing in patients with obstructive sleep apnea: a two-arm, prospective, randomized controlled trial
Source: BMC Anesthesiol. 2023 Jul 13;23:236. doi: 10.1186/s12871-023-02193-y (PMC10339502; doi:10.1186/s12871-023-02193-y)
Supplement: Supplementary file 1 — Additional file 1. [file 12871_2023_2193_MOESM1_ESM.docx]

**SUPPLEMENTARY MATERIAL**

**Supplementary Table 1. Patient demographics across the two groups (Intention to treat).**

|  | **Control** | **Intervention** | **P value** | **Standardized Mean Difference** |  |
| --- | --- | --- | --- | --- | --- |
| N | **n=20** | **n=15** |  |  |  |
| **Age** | 64 ± 11.3 | 66 ± 11.3 | 0.61 | 0.185 |  |
| **BMI** | 34.1 ± 5.3 | 34.4 ± 7.3 | 0.88 | 0.052 |  |
| **Neck Circumference** | 42.2 ± 3.2 | 41.6 ± 3.4 | 0.63 | 0.167 |  |
| **Gender F/M** | 11/9 | 8/7 | 1.00 | 0.033 |  |
| **STOP-Bang score** | 4.5 ± 1.1 | 4.5 ± 1.3 | 0.93 | 0.028 |  |
| **Co-morbidity** |  |  |  |  |  |
| Hypertension | 11 | 8 | 1.00 | 0.033 |  |
| Gastroesophageal reflux | 6 | 3 | 0.77 | 0.232 |  |
| Diabetes Mellitus | 2 | 5 | 0.11 | 0.591 |  |
| Smoker | 2 | 2 | 1.00 | 0.104 |  |
| Asthma | 3 | 1 | 0.62 | 0.271 |  |
| COPD | 2 | 0 | 0.50 | 0.471 |  |
| CAD | 3 | 0 | 0.24 | 0.594 |  |
|  |  |  |  |  |  |
| **Type of surgery** |  |  | 0.92 | 0.516 |  |
| Orthopedic | 12 | 8 |  |  |  |
| General | 6 | 5 |  |  |  |
| Gyn+Obsterics | 1 | 1 |  |  |  |
| Urology | 1 | 0 |  |  |  |
| **Type of Anesthesia** |  |  | 1.00 | 0.067 |  |
| General | 10 | 7 |  |  |  |
| Spinal | 10 | 8 |  |  |  |
| **ASA Status** |  |  | 0.74 | 0.149 |  |
| II | 9 | 6 |  |  |  |
| III | 10 | 9 |  |  |  |
| **Total amount of opioids (in mg IV morphine equivalents)** |  |  |  |  |  |
| 1^st^ 24 h | 10.3 [4.8 - 19.4] | 11.0 [6.3 – 20.8] | 0.83 | 0.166 |  |
| 1^st^ 48 h | 21.4 [13.8 - 41.8] | 37.5 [17.3 - 57.3] | 0.38 | 0.311 |  |
| 1^st^ 72 h | 28.8 [16.8 - 51.5] | 62.0 [23.1 - 82.6] | 0.28 | 0.397 |  |
| **Preoperative sleep study data between the two groups** | | | | | |
| AHI | 18.70 ± 14.20 | 21.87 ± 11.68 | 0.49 | 0.243 |  |
| OAI | 7.39 ± 7.83 | 12.15 ± 7.39 | 0.08 | 0.626 |  |
| OAHI | 16.02 ±10.57 | 21.23 ± 11.35 | 0.17 | 0.475 |  |
| CAI | 2.83 ± 8.00 | 0.65 ± 1.27 | 0.31 | 0.380 |  |
| HI | 8.63 ± 4.65 | 9.08 ± 8.48 | 0.84 | 0.065 |  |
| ODI | 15.5 [11.6 – 25.8] | 18.2 [13.8 – 33.8] | 0.48 | 0.219 |  |
| CT90 | 0.92 [0.15 – 3.33] | 1.92 [0.62 – 7.38] | 0.10 | 0.626 |  |
| Average SaO2 | 93.36 ± 1.51 | 93.09 ± 2.56 | 0.71 | 0.125 |  |
| Lowest SaO2 | 82.70 ± 5.34 | 78.07 ± 5.91 | 0.02 | 0.823 |  |

. Standardized Mean Difference was calculated using R package tableone

BMI - body mass index; COPD – chronic obstructive pulmonary disease; CAD – coronary artery disease; OR – operating room; h: hours.

AHI: Apnea-Hypopnea Index; Apnea index: average number of apnea episodes per hour; Arousal \index: number of arousals × 60 / Total sleep time; CAI: Central Apnea Index: total number of central apneas per hour; CT90: cumulative percentage of Total sleep time with oxygen desaturation below 90%; HI: Hypopnea index, average number of hypopnea episodes per hour; OAHI: Obstructive Apnea Hypopnea Index, total number of obstructive apneas and hypopneas per hour; OAI: Obstructive Apnea Index: total number of obstructive apneas divided by Total sleep time; ODI: Oxygen Desaturation Index, number of events with oxygen desaturation below 4% threshold in one hour; REM%: Time spent in rapid eye movement stage of sleep; SaO_2_: saturation of oxygen in hemoglobin

**Supplementary Table 2. Comparison of Preoperative and Postoperative sleep-related outcomes between groups (Intention to Treat)**

|  | Control (n = 20) | |  | Intervention (n = 15) | |  | Between group comparison (ANCOVA) |
| --- | --- | --- | --- | --- | --- | --- | --- |
| **Variable** | Pre-op | Post-op | p-value | Pre-op | Post-op | p-value | p-value |
| AHI | 18.7 ± 14.2 | 27.0 ± 27.0 | 0.41 | 21.9 ± 11.7 | 19.7 ± 23.5 | 0.30 | 0.17 |
| OAI | 7.4 ± 7.8 | 14.7 ± 17.0 | 0.10 | 12.1 ± 7.4 | 7.9 ± 11.4 | 0.12 | 0.03 |
| CAI | 2.8 ± 8.0 | 2.5 ± 4.3 | 0.71 | 0.6 ± 1.3 | 1.7 ± 4.1 | 0.78 | 0.61 |
| HI | 8.6 ± 4.6 | 10.3 ± 10.7 | 0.84 | 9.1 ± 8.5 | 10.0 ± 10.1 | 0.49 | 0.90 |
| OAHI | 16.0 ± 10.6 | 25.0 ± 24.2 | 0.10 | 21.2 ± 11.3 | 17.9 ± 20.2 | 0.36 | 0.06 |
| ODI | 20.3 ± 13.5 | 26.9 ± 25.0 | 0.28 | 23.3 ± 13.8 | 25.1 ± 27.6 | 0.93 | 0.49 |
| CT90 | 2.0 ± 2.4 | 22.9 ± 30.9 | 0.001 | 4.7 ± 5.6 | 17.7 ± 22.4 | 0.08 | 0.46 |
| Average SaO2 | 93.4 ± 1.5 | 90.5 ± 3.2 | 0.001 | 93.1 ± 2.6 | 89.8 ± 4.3 | 0.01 | 0.66 |
| Lowest SaO2 | 82.7 ± 5.3 | 77.2 ± 18.0 | 0.3 | 78.1 ± 5.9 | 75.9 ± 11.0 | 0.84 | 0.79 |
| Supine % | 48.0 ± 32.3 | 79.8 ± 29.4 | 0.004 | 37.8 ± 34.4 | 67.3 ± 44.0 | 0.03 | 0.81 |

AHI: Apnea-Hypopnea Index; Apnea index: average number of apnea episodes per hour; CAI: Central Apnea Index: total number of central apneas per hour; CT90: cumulative percentage of TST with oxygen desaturation below 90%; HI: Hypopnea index, average number of hypopnea episodes per hour; OAHI: Obstructive Apnea Hypopnea Index, total number of obstructive apneas and hypopneas per hour; OAI: Obstructive Apnea Index: total number of obstructive apneas divided by TST; ODI: Oxygen Desaturation Index, number of events with oxygen desaturation below 4% threshold in one hour; SaO2: saturation of oxygen in hemoglobin.

**Supplementary Figures**

| 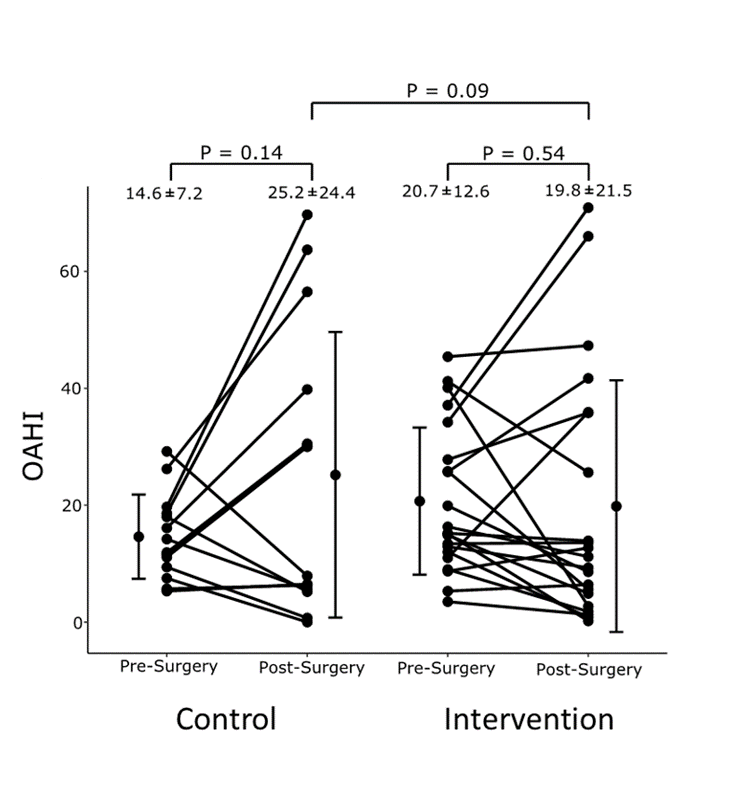   \| **Supplementary figure 1**. The effect of semi-upright position on obstructive apnea-hypopnea index in the two groups, by performing a per-protocol analysis. \| \| --- \|   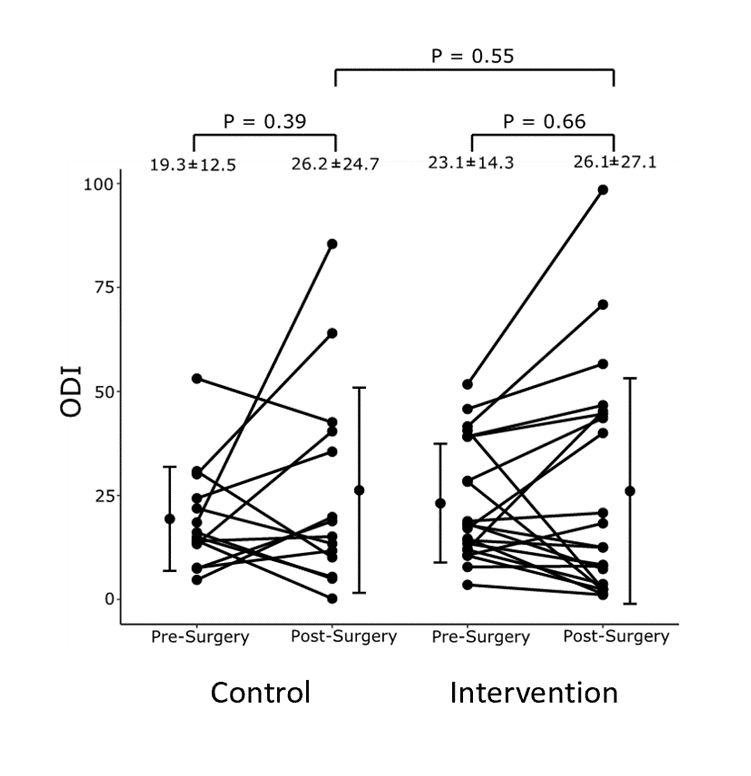 |
| --- | --- |
| **Supplementary figure 2**. The effect of semi-upright position on ODI in the two groups, by performing a per-protocol analysis. |

| 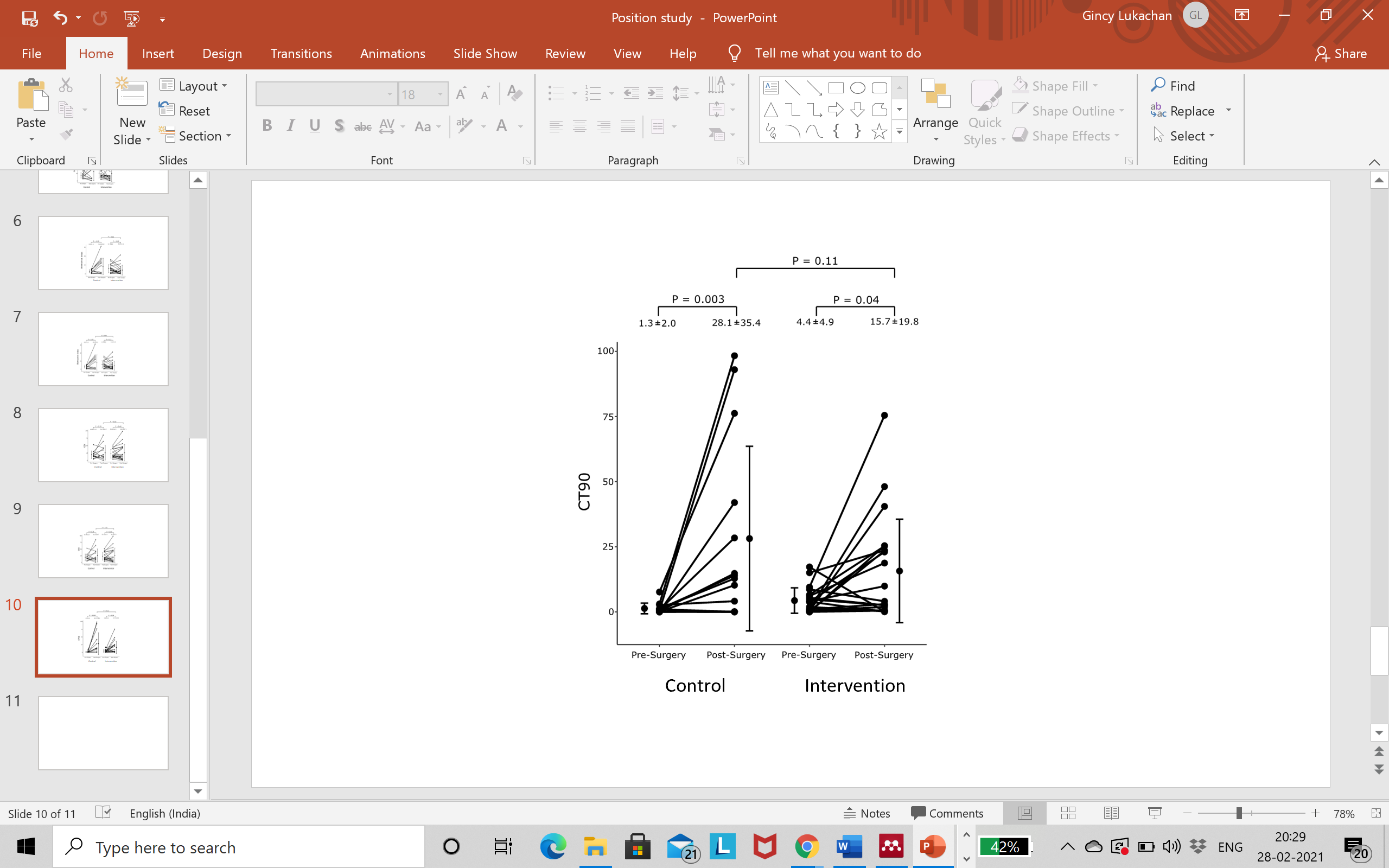 |
| --- |
| **Supplementary figure 3**. The effect of semi-upright position on CT90 in the two groups, by performing a per-protocol analysis. |

|  |
| --- |
|  |


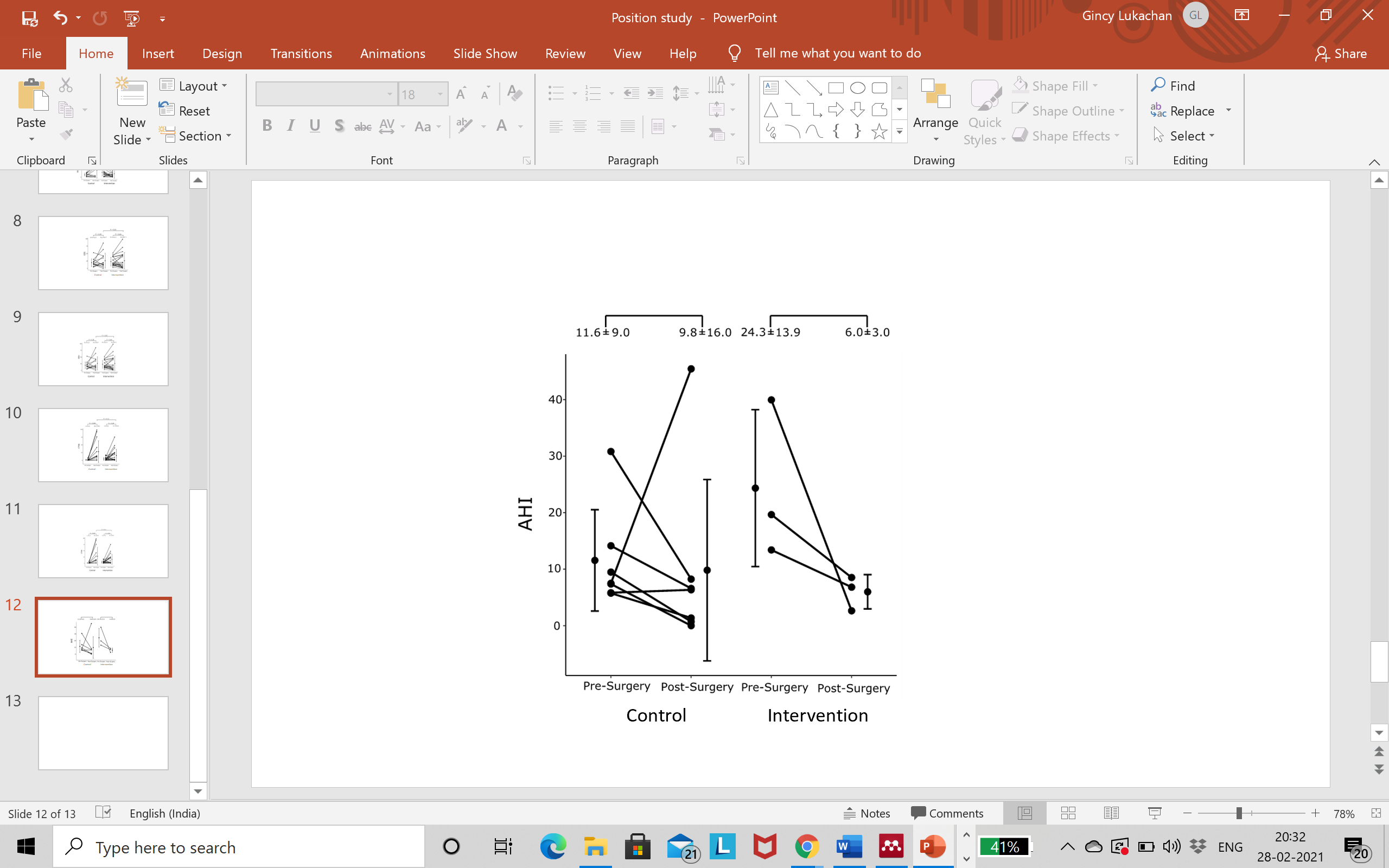


|  |
| --- |
| **Supplementary figure 4.** The effect of semi-upright body position on the apnea-hypopnea index (AHI in patients with supine-related OSA (n=10) |
